# Using 4D Cardiovascular Magnetic Resonance Imaging to Validate Computational Fluid Dynamics: A Case Study

**DOI:** 10.3389/fped.2015.00107

**Published:** 2015-12-14

**Authors:** Giovanni Biglino, Daria Cosentino, Jennifer A. Steeden, Lorenzo De Nova, Matteo Castelli, Hopewell Ntsinjana, Giancarlo Pennati, Andrew M. Taylor, Silvia Schievano

**Affiliations:** ^1^Centre for Cardiovascular Imaging, UCL Institute of Cardiovascular Science, Great Ormond Street Hospital for Children, NHS Foundation Trust, London, UK; ^2^Laboratory of Biological Structures Mechanics (LAbS), Politecnico di Milano, Milan, Italy

**Keywords:** cardiovascular magnetic resonance imaging, mock circulatory loop, validation, congenital heart disease, rapid prototyping

## Abstract

Computational fluid dynamics (CFD) can have a complementary predictive role alongside the exquisite visualization capabilities of 4D cardiovascular magnetic resonance (CMR) imaging. In order to exploit these capabilities (e.g., for decision-making), it is necessary to validate computational models against real world data. In this study, we sought to acquire 4D CMR flow data in a controllable, experimental setup and use these data to validate a corresponding computational model. We applied this paradigm to a case of congenital heart disease, namely, transposition of the great arteries (TGA) repaired with arterial switch operation. For this purpose, a mock circulatory loop compatible with the CMR environment was constructed and two detailed aortic 3D models (i.e., one TGA case and one normal aortic anatomy) were tested under realistic hemodynamic conditions, acquiring 4D CMR flow. The same 3D domains were used for multi-scale CFD simulations, whereby the remainder of the mock circulatory system was appropriately summarized with a lumped parameter network. Boundary conditions of the simulations mirrored those measured *in vitro*. Results showed a very good quantitative agreement between experimental and computational models in terms of pressure (overall maximum % error = 4.4% aortic pressure in the control anatomy) and flow distribution data (overall maximum % error = 3.6% at the subclavian artery outlet of the TGA model). Very good qualitative agreement could also be appreciated in terms of streamlines, throughout the cardiac cycle. Additionally, velocity vectors in the ascending aorta revealed less symmetrical flow in the TGA model, which also exhibited higher wall shear stress in the anterior ascending aorta.

## Introduction

Gathering insight into local hemodynamics of patients with congenital heart defects is crucial not only for improving general understanding of the physiology of such diseases, often associated with complex anatomies and intricate “plumbing,” but also for refining assessment of individual patients. In this context, the role of cardiovascular magnetic resonance (CMR) imaging is unquestionable, and four dimensional phase-contrast magnetic resonance (4D PCMR) flow imaging, in particular, has been shown to provide exquisite data. For example, 4D PCMR has been proven helpful in assessing systemic-to-pulmonary collateral flow in Fontan physiology (1) or evaluating blood flow characteristics after repair of tetralogy of Fallot (2, 3). This imaging technique, providing a 3D flow map of the blood circulation, can replace the attempts of computational fluid dynamics (CFD) simulations to explain complex hemodynamic scenarios. However, no matter how refined the imaging data, 4D PCMR lacks predictive capabilities, i.e., simulating multiple scenarios for the same patient. On top of this, this type of acquisition is still relatively long (~15 min), especially for a younger population, despite efforts in accelerating 4D sequences (4). Furthermore, not all centers are yet proficient in using this technique, which – at present – is not part of routine protocols but rather used for *ad hoc* cases or research studies. This is where CFD analyses can have a complementary, and potentially clinically relevant, role.

Computational fluid dynamics possesses predictive capabilities, whereby different scenarios can be tested parametrically, even at a patient-specific level (5–7). Its potential in assessing complex congenital scenarios has been extensively discussed in the literature (8, 9). Nevertheless, in order for computational models to be potentially integrated into clinical practice or used to inform clinicians, e.g., for decision-making, such models must be validated. Validation is a process, whereby the accuracy of a computational model is assessed against real world data (10). In the present study, we discuss how an experimental model can be used for validating a computational model of a complex case of congenital heart disease, namely, transposition of the great arteries (TGA) repaired with arterial switch operation (ASO) and Lecompte maneuver (11). Particular attention is given to the role of CMR in this context, which is indeed multifaceted. In this study, CMR was used for

- reconstruction of anatomies from 3D wholeheart and creation of 3D patient-specific models (i.e., CMR provides anatomical information),
- setting up the model with patient-specific values and definition of appropriate boundary conditions (i.e., CMR provides functional data), and
- validating a CFD model both qualitatively (i.e., streamlines) and quantitatively (i.e., flow-velocity and flow distribution).

## Materials and Methods

### Experimental Model

**Figure 1:**
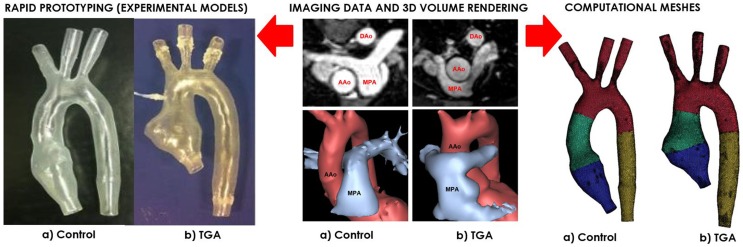
**Both control (a) and TGA (b) anatomies are generated from CMR data (*center*)**. The exact same 3D volumes are manufactured with rapid prototyping (*left*) and meshed for computational simulations (*right*). Please note typical features of TGA anatomy, particularly the enlarged aortic root with a visible protrusion. AAo = ascending aorta; DAo = descending aorta; MPA = main pulmonary artery.

A patient-specific model of aortic arch was generated for insertion in a CMR-compatible mock circulatory system. The anatomy was derived from the CMR examination (3D wholeheart dataset) of a 15-year-old male patient with repaired TGA (Figure 1). In order to appreciate features specific to TGA, with its typical arrangement of the great vessels, i.e., enlarged aortic root and main pulmonary artery straddling the aorta following the Lecompte maneuver, an age-matched healthy male subject was also included in the study. The latter came to our center for the assessment of hereditary cardiomyopathy and proved negative on CMR and genetic screenings. In both cases, Institutional ethical approval was in place for research use of CMR data.

Three-dimensional volumes of the aortic root, aortic arch, head-and-neck vessels, and descending aorta were generated from the 3D wholeheart using commercial software (Mimics, Materialise, Leuven, Belgium), as previously described in Ref. (12). The models were printed by means of rapid prototyping technology using a robust and transparent rigid resin (Watershed 11122, DSM Somos, Elgin, IL, USA), with an arbitrary wall thickness of 1.5 mm to ensure robustness.

**Figure 2:**
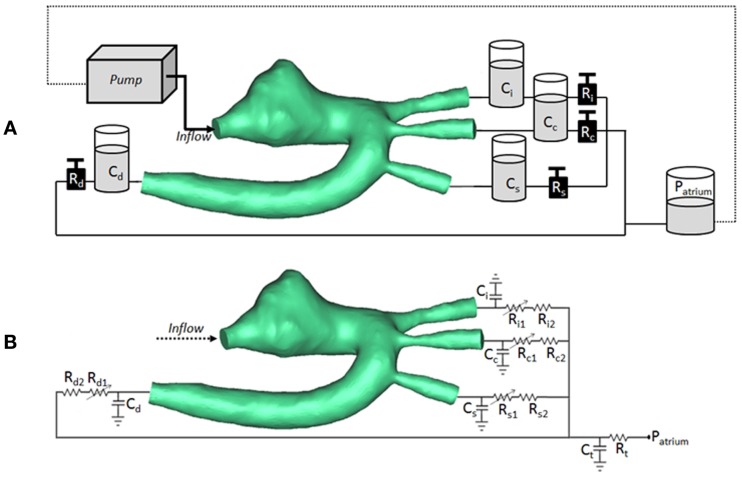
**(A)** Arrangement of compliant (C) and resistive (R) elements in the *in vitro* setup, as air chambers and metered taps, respectively, also showing the 3D rapid-prototyped model (TGA patient in the picture), the pump, and the atrial chamber implementing atrial pressure (*P*_atrium_). **(B)** Arrangement for corresponding multi-scale CFD simulation, including 3D volume of TGA patient, coupled with lumped parameter network summarizing the remainder of the system. Each outlet is simulated with a non-linear and linear resistor in series. *C*_i_/*R*_i_ = compliance/resistance for innominate artery; *C*_c_/*R*_c_ = compliance/resistance for carotid artery; *C*_s_/*R*_s_ = compliance/resistance for subclavian artery; *C*_d_/*R*_d_ = compliance/resistance for descending aorta; *C*_t_/*R*_t_ = terminal compliance/resistance; *P*_atrium_ = atrial pressure.

The CMR-compatible mock circulatory loop (Figure 2A) for *in vitro* acquisitions of 4D PCMR data consisted of the following components:

(a) pulsatile pump (Harvard Apparatus, Holliston, MA, USA) for generating adequate flow waveform;
(b) braided pipes from and to the pump;
(c) patient-specific 3D phantom, including side-port for insertion of pressure sensor in the aortic arch;
(d) resistive (R) elements in the form of metered and calibrated needle-pinch valves;
(e) compliance (C) elements in the form of Windkessel chambers with adjustable air volume;
(f) atrial reservoir implementing a constant head pressure (i.e., atrial pressure) of 8 mmHg;
(g) silicone tubings connecting the outlets of the 3D models, ultimately merging to the braided outflow pipe feeding back to the pump.

For both TGA and control models, pressure data were recorded during CMR acquisition inside the aortic arch using a high-fidelity pre-calibrated fiber-optic pressure sensor (Preclin, Samba sensors AB, Vastra Frolunda, Sweden), whose calibration was checked using the method of column of water prior to the tests. Flow information on stroke volume (SV) and flow waveform shape were recorded during CMR acquisition using a custom-made MR-compatible ultra-sonic flow probe (9PXL probe, Transonic Inc., Ithaca, NY, USA) connected to a flow meter (400-Series Multi-Channel Flowmeter Consoles and Modules for Laboratory Research, Transonic Inc,). The probe was calibrated with the method of timed collection prior to the experiments.

The data acquisition system (BIOPAC System Inc., Goleta, CA, USA) was connected to a laptop for visualization and analysis of the traces. Data were recorded at 250 Hz (AcqKnowledge 4.1.1, BIOPAC System Inc.).

### Cardiovascular Magnetic Resonance Imaging

Once inside the scanner (1.5 T Avanto; Siemens Medical Systems, Erlangen, Germany), and having verified the absence of leaks and bubbles in the system, CMR acquisitions were performed.

Acquisitions were gated to the pump external trigger, via a BNC connection cable.

2D retrospectively gated phase-contrast Cartesian flows were acquired at different locations along the model at the following positions: aortic root, ascending aorta, brachiocephalic branches (innominate, left carotid, left subclavian), and descending aorta. Settings for these acquisitions were VENC = 500 cm/s for the inlet, 250 cm/s for the outlets; echo time = 2.18 ms; temporal resolution = 29.9 ms; pixel spacing = 1.17 mm; slice thickness = 5 mm; flip angle = 30°. 4D PCMR acquisitions were performed using a prospectively gated Cartesian sequence with the following settings: VENC = 200 cm/s; echo time = 2.5 ms; temporal resolution = 33.4 ms; pixel spacing = 2.2 mm × 2.2 mm; slice thickness = 2.2 mm; flip angle = 5°. Scanning time for 4D flow was ~15 min. Post-processing of the 2D PCMR data was carried out using in-house written plugins for the DICOM viewer OsiriX and, for the 4D flow data, using commercial visualization software (4D Flow v.2.4, Siemens).

### Computational Fluid Dynamics

The exact same anatomical models studied *in vitro* were used for numerical simulations using a multi-scale, or multi-domain, approach (13, 14), whereby such 3D volumes were coupled with a lumped parameter network (LPN) summarizing the remainder of the circulation in the experimental setup. The steps for making the computational model are reported here in detail.

Computational meshes were created with ICEM (Ansys Inc., Canonsburg, PA, USA) based on the exact same .stl files as for 3D printing the anatomical models, adopting tetrahedral elements. Additionally, a wall mesh inflation (5 layers of prisms, 1.2 growth ratio, and 1.0 mm maximum height) was applied in order to efficiently resolve boundary layer flows. Five different element sizes were used resulting in meshes ranging from 400,000 to 1,200,000 volumes. A mesh sensitivity analysis was conducted to identify the best compromise between accuracy of the results and computational time required by the CFD simulation to converge. The influence of the mesh size was evaluated on the calculated power dissipation index (15, 16), showing a negligible difference of 1.4% (corresponding to 1⋅104 W) between the mesh with 900,000 and 1,200,000 elements; thus, the former mesh was adopted for the analyses. The commercial finite volume software Ansys Fluent (Ansys, Fluent Inc.©, Lebanon, NH, USA) was used to set and run all the CFD simulations. A second order upwind scheme was used for the solution of the Navier-Stokes momentum equations, with a standard spatial discretization for the pressure, and an implicit least-square cell-based discretization for the gradient. A semi-implicit method for pressure linked equations (SIMPLE) pressure–velocity coupling algorithm was exploited. The under-relaxation factors were set as default to 0.3 for the pressure and 0.7 for the momentum. The absolute convergence criterion was the residuals of mass and momentum conservation equations to be <10^−4^.

In order to replicate the *in vitro* conditions, the fluid used in the simulations was water (incompressible, Newtonian, density ρ = 1000 kg/m^3^, and viscosity μ = 1 cP). The simulations were run under the hypothesis of laminar flow, no gravitational effects, isothermic conditions, rigid walls, and no-slip conditions.

At the inlet of the model, a time-varying velocity function interpolating the experimental 2D PCMR flow curve was imposed through a user-defined function (UDF) script on the whole inlet plane. The LPN representing the experimental circuit is summarized in Figure 2B.

The settings at which the acquisitions were performed are summarized in Table 1. The values of the resistances and the compliances were chosen in order to replicate the respective components of the experimental circuit. The non-linear resistance *R*_1_ corresponds to the taps of the mock circuit and is characterized through the pressure drop-flow relationship obtained during the tap experimental calibration. The other resistances included in the network take into account the distributed and the concentrated pressure drops due to the length of the pipes, the presence of connections, and the sudden variations in diameter at different sections. In particular, the linear resistance *R*_2_ represents the pressure drop caused by the connections of the tubes, while *R*_t_ lumps the final part of the tube connecting all outlets to the right atrium reservoir.

**Table 1:** **Value of the parameters chosen to characterize each downstream branch j: non-linear resistances indicated through the pressure drop (Δ*P*) across the resistance *R*_j1_ (with *Q* indicating the volumetric flow-rate); linear resistances *R*_j2_ to account for the distributed resistances; compliances *C*_j_**.

Branch	*R*_j1_	*R*_j2_	*C*_j_
	Δ*P* (Pa)	(Pa⋅s/m^3^)	(m^3^/Pa)
Innominate (j = *i*)	1.10^13^⋅*Q*^2^–8.10^6^⋅*Q*	5⋅10^7^	1.88⋅10^−9^
Carotid (j = *c*)	2.10^13^⋅*Q*^2^–5.106⋅*Q*	1⋅10^8^	1.98⋅10^−9^
Subclavian (j = *s*)	1.10^13^⋅*Q*^2^–8.106⋅*Q*	1⋅10^7^	3.06⋅10^−9^
Descending aorta (j = *d*)	8.10^11^⋅*Q*^2^–1.107⋅*Q*	2.5⋅10^7^	1.48⋅10^−9^
Common section (j = *t*)	–	8⋅10^7^	0.36⋅10^−11^

The ordinary differential equations (ODEs) are resolved through the explicit Euler numerical method, using a time-step Δ*t* of 10^−4^ s.

### Data Analysis

Having ensured the realistic nature of the *in vitro* data, the focus of the study was ultimately to compare computational vs. experimental results. In this sense,

- mean flows were measured at all outlets,
- pressure was measured in the ascending aorta via the side-port and at the equivalent location in the computational model, and
- streamlines were assessed and compared (4D PCMR vs. CFD) to verify agreement in local fluid dynamics.

Data on the eccentricity of flow were also derived from velocity vectors at three planes (i.e., ascending aorta, aortic arch, and descending aorta) from CFD.

Finally, wall shear stress (τ_w_ = μ⋅∂*u*/∂*y*, where μ = fluid viscosity, *u* = velocity of the fluid along the boundary and *y* = distance to the wall, with *y* = 0 for τ_w_) was also calculated, as an additional output from the computational model.

Mean pressure and flow data were compared at set time intervals (0.1, 0.2, 0.4, and 0.6 s) throughout the cardiac cycle as indicative of early systole, peak systole, late systole, and diastole. Experimental values were reported as mean ± SD, whereas computational results were taken when convergence was reached.

## Results

**Figure 3:**
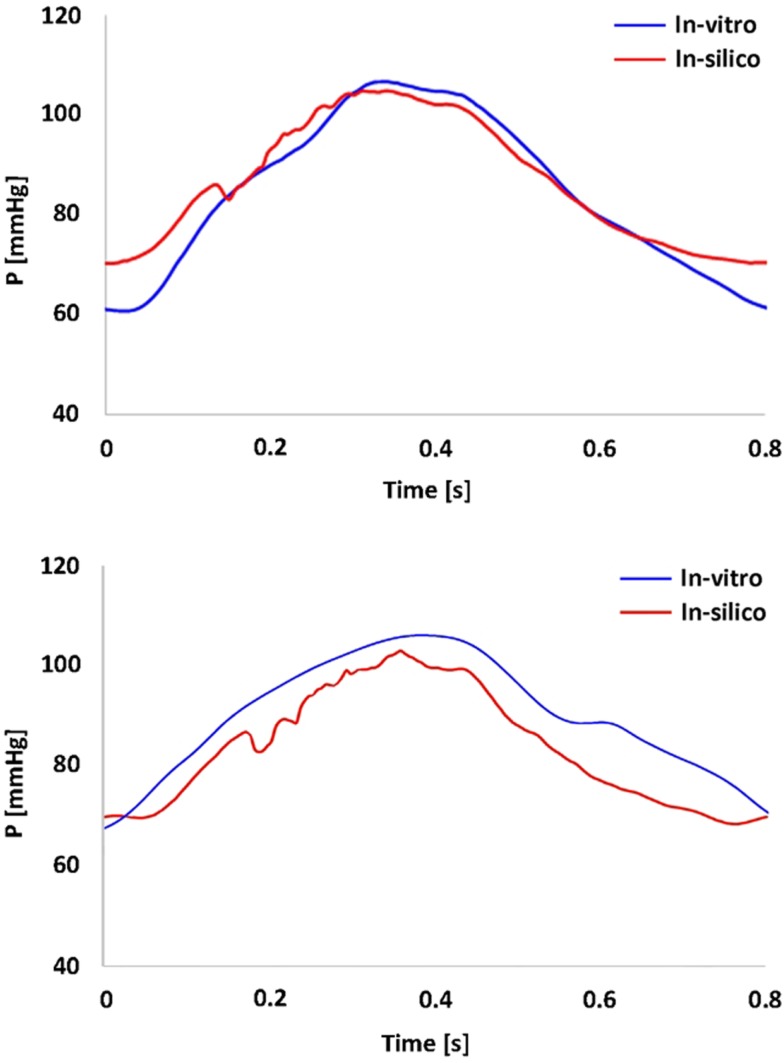
**Summary of pressure data showing the realistic shape and range of the pressure waveform gathered in the TGA model (*top panel*) and in the control model (*bottom panel*)**. *In vitro* (*blue*) and *in silico* (*red*) signals are superimposed.

*In vitro* data were successfully acquired within the CMR- compatible mock circulatory loop, gathering 4D CMR images in both anatomical models. Experimental pressure signals were realistic in shape and within a physiological range (Figure 3), with low SD indicating good repeatability (peak systolic = 106 ± 0.7 mmHg and minimum diastolic = 60 ± 0.5 mmHg for the TGA model; peak systolic = 105 ± 0.5 mmHg and minimum diastolic = 66 ± 0.5 mmHg for the control model).

**Figure 4:**
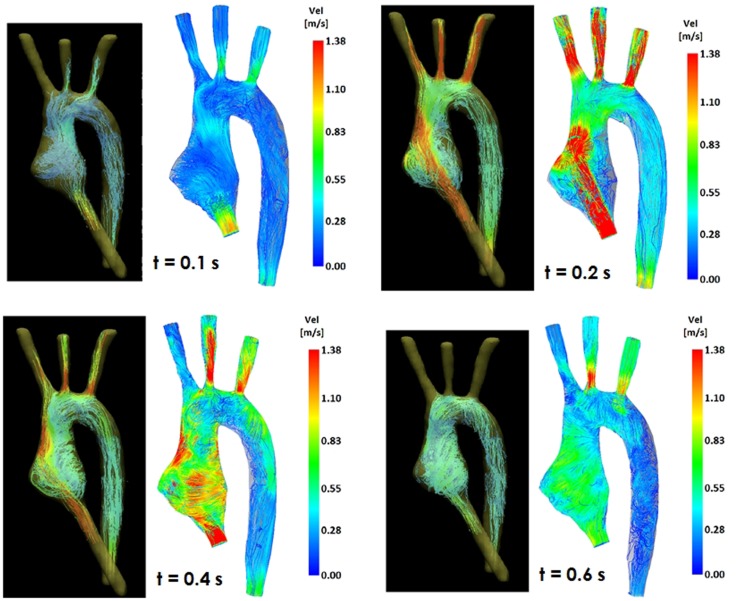
**Comparison of flow streamlines in the TGA 3D model, 4D CMR data (*left*) and CFD results (*right*), at four different time points in the cardiac cycle**.

**Figure 5:**
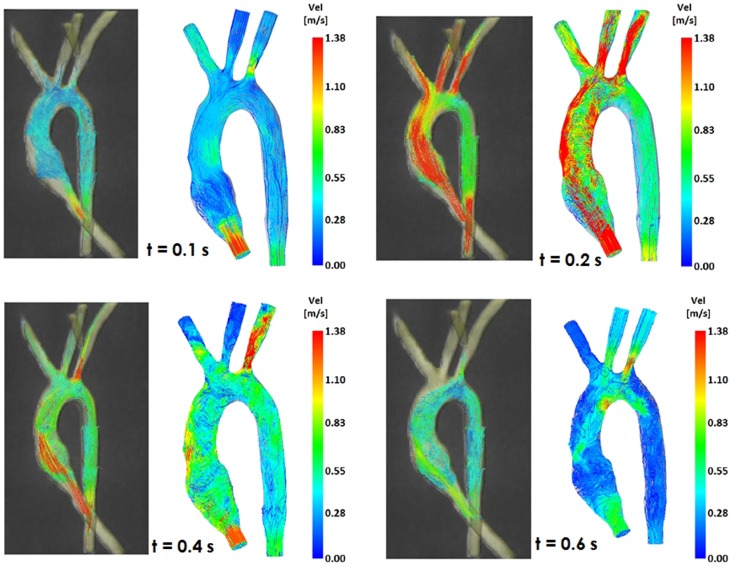
**Comparison of flow streamlines in the control 3D model, 4D CMR data (*left*) and CFD results (*right*), at four different time points in the cardiac cycle**.

**Figure 6:**
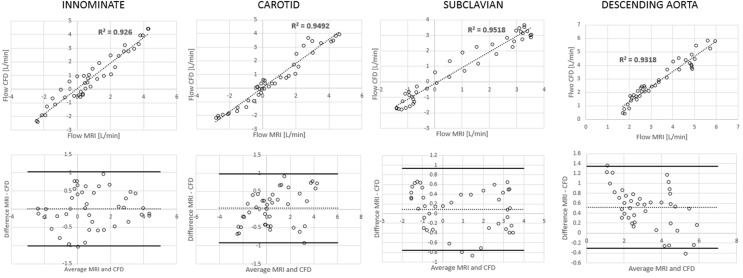
**Correlation and Bland Altman plots for flow measurements (CMR vs. CFD) at four locations in the control model**.

**Figure 7:**
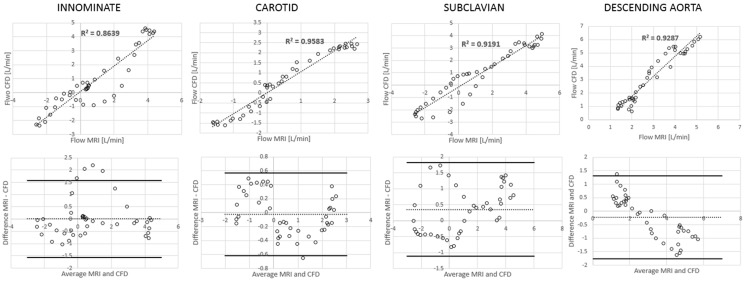
**Correlation and Bland Altman plots for flow measurements (CMR vs. CFD) at four locations in the TGA model**.

Comparing computational simulation against *in vitro* data, it was noted that pressure values as well as waveforms were in satisfactory agreement. In particular, the mean pressure measured experimentally in the TGA aortic arch was 84.6 vs. 85.7 mmHg from CFD analysis (1.3% difference). For the control model, the experimental mean aortic pressure was 87.0 mmHg, and the computational mean pressure value was 83.2 mmHg (4.4% difference). Mean flow values, flow distribution at the outlets of the model, as well as flow waveforms were also all in good agreement between experimental and computational results (Table 2). Furthermore, excellent qualitative agreement was verified between the CMR and the CFD data (Figures 4 and 5): the CFD simulation was able to reproduce the same flow jet impinging at the top of the TGA aortic root wall, and the surrounding whirls visible in the 4D flow images. The range of velocities is comparable in both magnitude and distribution. Excellent correspondence was noticed throughout the cardiac cycle. For both models, CMR and CFD flow measurements at four locations (innominate, subclavian, carotid, and descending aorta) showed a strong correlation, with *R*^2^ always >0.9, except for the innominate branch of the TGA model (*R*^2^ = 0.86), and additionally Bland Altman plots generally show a good agreement in flow measurements at all locations. These observations are summarized in Figures 6 and 7.

**Table 2:** **Flow distribution in L/min and as % flow split (in brackets) at all outlets of the model, comparing quantification from 2D PCMR and CFD results**.

Outlet	TGA model		Control model	
	CMR	CFD	CMR	CFD
	L/min (%)	L/min (%)	L/min (%)	L/min (%)
Innominate	0.92 (16.7)	0.86 (15.7)	0.85 (14.6)	0.88 (16.7)
Carotid	0.54 (9.9)	0.56 (10.3)	0.59 (10.2)	0.58 (11.1)
Subclavian	1.17 (21.4)	0.98 (17.8)	0.92 (15.9)	0.88 (16.9)
Descending aorta	2.92 (53.1)	3.02 (55.1)	3.39 (57.9)	2.91 (55.3)

**Figure 8:**
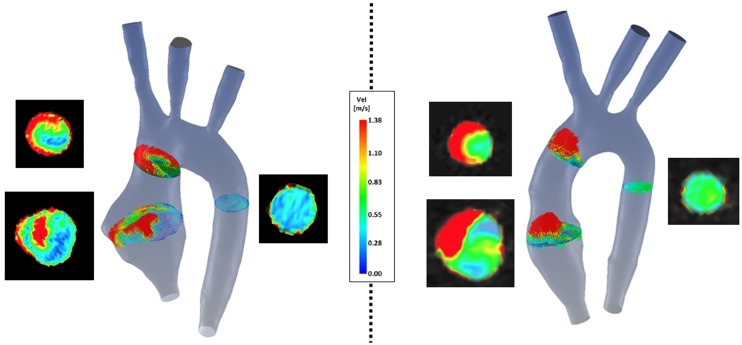
**Comparison of velocity vectors at three cross-sections along the aorta in both TGA (*left*) and control (*right*) anatomies**. The corresponding velocity data at the same planes extracted from the experimental 4D CMR are shown next to each model, showing good agreement on the same velocity scale.

**Figure 9:**
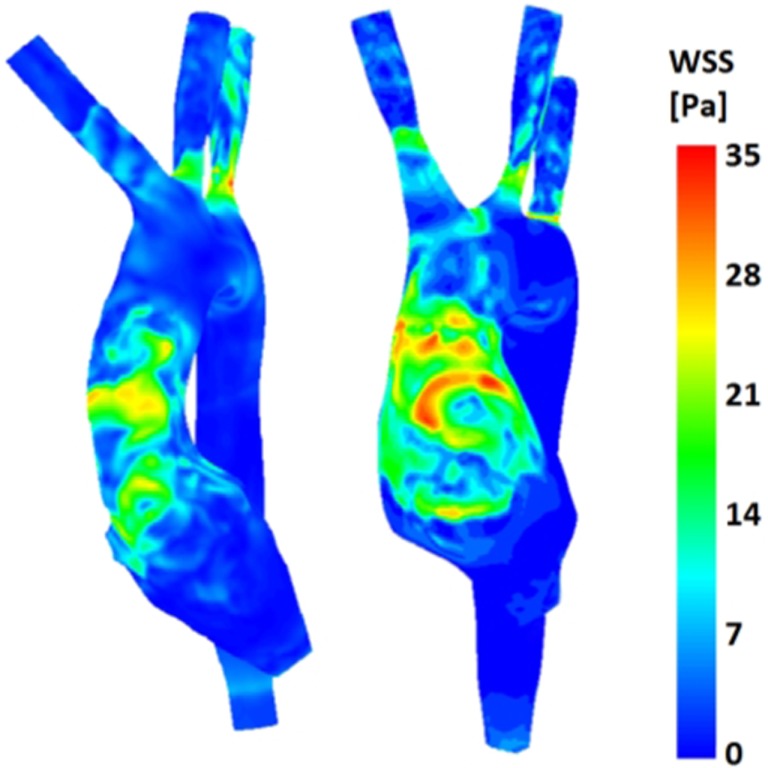
**Comparison of wall shear stress (WSS) distribution on the aortic wall for both control (*left*) and TGA (*right*) anatomies, highlighting higher WSS in anterior ascending aorta of the TGA model**.

Comparing TGA and control anatomies, no substantial difference in flow split was overall appreciated (Table 1). This result is not surprising, since R and C settings were purposefully kept constant for both acquisitions. The effect of geometrical features (particularly the enlarged aortic root and the more gothic shape of the arch) is instead clearly noticeable from local fluid dynamics, with differences in the streamlines especially visible in the aortic root at end systole. This comparison is further enriched with additional computational results:

(a) Symmetry (Figure 8): comparison of velocity vectors in the ascending aorta reveals an appreciably less symmetrical flow in the TGA model, with a skewed peak of flow velocity and presence of secondary flows. Higher velocities in the control model are clustered in the center of the root mid-sectional place, while in the TGA model they present a more random distribution.
(b) Wall shear stress (Figure 9): the TGA model presented a more extensive area of the ascending aorta with high τ_w_, with portions reaching values of 35 Pa.

## Discussion

This study makes use of experimental and computational approaches, assessing the fluid dynamics of repaired congenital heart disease, particularly repaired TGA with ASO, presenting a patient-specific example as well as an age-matched control case for reference. From an experimental standpoint, 4D CMR acquisitions were obtained in a CMR-compatible mock circulatory system. 4D flow data were then used to validate the corresponding computational model.

Satisfactory agreement was noted between experimental and computational results, with mean pressure and flow values, flow distribution at the different model outlets and flow streamlines all matching well between the two. Importantly, making use of 4D CMR has the additional benefit of providing local fluid dynamics information, which means that the computational model is not only sufficiently accurate to capture mean global phenomena (e.g., correct pressure values) but also local phenomena such as the noticeable vortex which develops in the enlarged TGA aortic root during systole. This, in turn, gives the user additional confidence in the robustness of the computational model, whose predictive capabilities can then be used more reliably, whereby different scenarios can be virtually simulated.

Very few studies have taken this parallel approach in testing CMR and CFD. In one case (17), the purpose of the study was indeed to compare results of 4D CMR and CFD in a simple *in vitro* setup and in more complex *in vivo* models of thoracic aorta, ultimately observing that overall the observed patterns were coherent. This study showed that by using correctly this methodological framework (e.g., patient-specific boundary conditions with fine boundary layer mesh), CFD can compute very accurate flow and vessel wall parameters, including wall shear stress (τ_w_). In fact, substantial differences in τ_w_ were observed in our case between TGA and control anatomies, suggesting that the effect of aortic arch morphology on local hemodynamics impacting in turn on the stresses experienced by the aortic wall, albeit in a rigid model. Our study, in agreement with Stalder et al. (17), shows that combining 4D CMR and CFD methodologies in a synergistic way can improve the understanding of *in vivo* hemodynamics. A very recent study (18) has applied the paradigm of modeling congenital heart disease *in vitro* and *in silico*, including 4D CMR, for an interesting case of Fontan circulation (total cavopulmonary connection). This study supports our observations that patient-specific models of congenital defects including 4D CMR allow fine tuning of CFD models, ultimately narrowing the gap for clinical implementation of the numerical models themselves.

Computationally, it is also possible to easily undertake parametric studies, by simply varying one parameter at a time in order to evaluate its influence on the fluid dynamics. The only concern would be represented by the additional computational cost of each simulation, rather than the (longer) time required to re-assemble an experimental rig. Another advantage of the computational model is the easy retrospective extraction of additional data, e.g., values of interest at different locations in the model, not necessarily planned beforehand, while this is not possible in an experimental study. These points depend crucially on validation of the numerical model itself.

With regards to τ_w_, differences can carry clinically meaningful information. From a numerical perspective, it should be, however, noted that this study made use of a rigid wall assumption, as a counterpart for an *in vitro* model fabricated using rigid resin, while small differences have been previously reported for time averaged τ_w_ and oscillatory shear index between rigid wall model and a fluid-structure interaction (FSI) models, larger differences were observed in terms of instantaneous τ_w_ (19). An FSI numerical model would therefore be preferable if instantaneous τ_w_ was a main output of interest, and in this case, the experimental counterpart of the study should involve a deformable model. Options for compliant arterial models have been discussed and are currently being explored (20). While taking advantage of a compliant model with realistic distensibility would be desirable to get any clinically relevant insight, this study is rather focused on methodological considerations on how to setup an *in vitro*/*in silico* comparison relevant for a hemodynamic scenario of congenital heart disease, including use of 4D CMR. It should be also noted that an additional limitation of the present model is represented by absence of the aortic valve, whereas instead a cylindrical inlet (12.0 mm diameter) was attached to the anatomical model. Inclusion of a valve in the experimental model would again render necessary a FSI approach for the computational counterpart. While the absence of a valve would impact of the physiological nature of measured flow features, the focus of the study was a comparison between experimental and computational data. Another improvement to the overall setup would be the employment of a more realistic flowing medium, such as a mixture of water and glycerine, which is a well-known analog for blood rheological properties.

While CMR can be prone to limitations in terms of spatial and temporal resolution, hence the duration of the acquisition, CFD results are affected by assumptions (e.g., flow regime) and boundary conditions. With regards to the latter and setting up the computational model, one further development that can be envisaged is the refinement of simulations by imposing a more detailed flow profile directly derived from 4D flow data, if available, rather than imposing a flow waveform across the designated inlet surface of the computational model. It would be interesting to explore in the future if this results in even more accurate information *in silico*.

Ultimately, evidence of the reliability of computational models is essential to take full advantage of the additional detailed local fluid dynamics information that can be extracted, particularly in simulated scenarios. By testing a patient-specific model *in vitro* and *in silico* and obtaining good agreement between the experimental data and the computational results, both in terms of overall fluid dynamics (e.g., % flow distribution) and local fluid dynamics (e.g., streamlines and vortices), then it is possible to take full advantage of the predictive capabilities of the computational model. This would imply reliably simulating a range of surgical scenarios (21) or a range of different devices implantation in the same anatomy, i.e., same patient, as described elsewhere in the literature (22).

## Conclusion

This study presented methodological considerations with regards to setting up a CMR-compatible mock circulatory system (incorporating patient-specific 3D preliminary anatomical models) for comparison with CFD modeling. While this is a preliminary study not intended to draw any clinical conclusion with regards to TGA hemodynamics, it demonstrates the feasibility of the method in the context of congenital heart disease, as good agreement was achieved between numerical simulations and experimental data acquired in the CMR setting. 4D CMR still requires manipulation of very large datasets and is not performed routinely in all clinical centers. Validated CFD models could be exploited for predictive simulations and gathering additional insight into the local fluid dynamics of congenital anatomies.

## Author Contributions

GB was involved in study design, processing medical images, data acquisition and processing, and drafting the manuscript; DC was involved in study design, computational simulations, and data processing; JS oversaw the acquisition of magnetic resonance imaging data and verified the manuscript’s contents; LN and MC were involved in acquiring and analyzing the data, as well as checking the manuscript content; HN was involved in study design, data acquisition, and checking the manuscript’s contents; GP was involved in data analysis and checking the manuscript’s contents; AT and SS were involved in designing the study, interpreting the data, and verifying the manuscript’s contents.

## Conflict of Interest Statement

The authors declare that the research was conducted in the absence of any commercial or financial relationships that could be construed as a potential conflict of interest.
